# Trichothecin Induces Cell Death in NF-κB Constitutively Activated Human Cancer Cells via Inhibition of IKKβ Phosphorylation

**DOI:** 10.1371/journal.pone.0071333

**Published:** 2013-08-01

**Authors:** Jia Su, Peiji Zhao, Lingmei Kong, Xingyao Li, Juming Yan, Ying Zeng, Yan Li

**Affiliations:** 1 State Key Laboratory of Phytochemistry and Plant Resources in West China, Kunming Institute of Botany, Chinese Academy of Sciences, Kunming, China; 2 University of the Chinese Academy of Sciences, Beijing, China; Westmead Millennium Institute, University of Sydney, Australia

## Abstract

Constitutive activation of the transcription factor nuclear factor-κB (NF-κB) is involved in tumorigenesis and chemo-resistance. As the key regulator of NF-κB, IKKβ is a major therapeutic target for various cancers. Trichothecin (TCN) is a metabolite isolated from an endophytic fungus of the herbal plant *Maytenus hookeri Loes.* In this study, we evaluated the anti-tumor activity of TCN and found that TCN markedly inhibits the growth of cancer cells with constitutively activated NF-κB. TCN induces G0/G1 cell cycle arrest and apoptosis in cancer cells, activating pro-apoptotic proteins, including caspase-3, -8 and PARP-1, and decreasing the expression of anti-apoptotic proteins Bcl-2, Bcl-xL, and survivin. Reporter activity assay and target genes expression analysis illustrated that TCN works as a potent inhibitor of the NF-κB signaling pathway. TCN inhibits the phosphorylation and degradation of IκBα and blocks the nuclear translocation of p65, and thus inhibits the expression of NF-κB target genes XIAP, cyclin D1, and Bcl-xL. Though TCN does not directly interfere with IKKβ kinase, it suppresses the phosphorylation of IKKβ. Overexpression of constitutively activated IKKβ aborted TCN induced cancer cell apoptosis, whereas knockdown of endogenous IKKβ with siRNA sensitized cancer cells toward apoptosis induced by TCN. Moreover, TCN showed a markedly weaker effect on normal cells. These findings suggest that TCN may be a potential therapeutic candidate for cancer treatment, targeting NF-κB signaling.

## Introduction

NF-κB transcription factors consist of five homologous subunits: RelA (p65), RelB, cRel (Rel), NF-κB1 (p50 and its precursor p105) and NF-κB2 (p52 and its precursor p100), which function as various homodimers and heterodimers [1], [2]. In the canonical NF-κB pathway, cells can be stimulated by different stimuli, including reactive oxygen species, tumor necrosis factor alpha, interleukin 1-beta, bacterial lipopolysaccharides, etc. Upon activation, the inhibitory subunit IκBα is phosphorylated by the IκB kinase (IKK) complex, which is then ubiquitinated and degraded through the proteasome pathway, promoting translocation of the p65/p50 complex into the nucleus and activating the expression of downstream genes [3], [4].

NF-κB signaling plays an important role in regulating inflammation, tumorigenesis and cancer development [5]–[7]. In a wide variety of cancers–including hematogenous malignancies (such as leukemia, lymphoma, and multiple myeloma), and solid tumors (such as lung, breast and pancreas)–NF-κB is persistently activated [8], [9]. Activation of NF-κB up-regulates the expression of anti-apoptotic genes encoding Bcl-xL, XIAP, cIAP1 and cIAP2, as well as proliferative genes such as cyclin D1 and IL-6 [10]–[13]. NF-κB activity is also closely connected to tumor metastasis and cancer chemo-resistance. NF-κB activation induces the transcription of genes involved in angiogenesis, a critical process in tumor formation and metastasis [14]. Moreover, NF-κB inhibitors enhance sensitivity of cancers to chemotherapeutic agents, such as paclitaxol, TNF-α and TRIAL [15]–[17]. Given the connection between NF-κB and cancer, the development of NF-κB inhibitor holds great potential in suppressing certain types of cancer proliferation as well as improving existing cancer therapies [18], [19].

*Maytenus hookeri Loes.* has been used as a folk remedy for a long time in southwest China because of its anticancer and anti-inflammatory activities. Previously, maytansine was identified for its anticancer effect by interfering microtubules [20], [21]. The derivative of maytansine, DM1, has been used in trastuzumab emtansine (T-DM1), a novel drug developed for treatment of HER2-positive breast cancer [22]. However, the chemical constituents responsible for the anticancer activities of this plant deserve further exploration.

Trichothecin (TCN) is isolated from the endophytic fungus of *Maytenus hookeri Loes*. Previous reports of ours and others demonstrated that TCN is involved with some anti-tumor activities, but the anti-tumor profiling or underlying mechanisms of these actions are lacking [23]–[25]. In the present study, we found that TCN inhibits the growth of human cancer cells by inhibiting NF-κB signaling. Our data showed that TCN suppresses the activation of IKKβ by suppressing its phosphorylation, inhibits the expression of NF-κB target genes, and induces cell cycle arrest and cancer cell apoptosis. As a novel inhibitor of the NF-κB pathway, TCN may prove to be a potentially promising drug candidate in developing novel cancer therapeutics.

## Materials and Methods

### Cell Lines

Human cancer cell lines, HepG2, A549, PANC-1 and HL-60, human embryonic kidney cell line HEK 293T, human bronchial epithelial cell line BEAS-2B and human kidney proximal tubule epithelial cell line HK-2 were purchased from ATCC. Human colonic epithelial cell line (CCD-841-CoN) was kindly gifted by Dr Lin, Li of the Institute of Biochemistry and Cell Biology, Chinese Academy of Sciences (Shanghai, China) [26]. Cells were cultured in 5% CO_2_ at 37°C in RPMI-1640, MEM or DMEM media containing 10% (v/v) fetal bovine serum (HyClone, Logan, UT).

### Reagents

Monoclonal antibodies to NF-κB P65, PARP-1, caspase-8, Bcl-xL, caspase-3, cyclin D1, Bcl-2, survivin, phospho-IKKβ (Ser177), and β-actin as well as polyclonal antibodies to IκBα, phospho-IκBα (pS32/pS36) were purchased from Santa-Cruz Biotechnology (Santa Cruz, CA). The XIAP polyclonal antibody was obtained from Proteintech (Chicago, IL). Monoclonal antibody to phospho-NF-κB p65 and polyclonal antibody to total IKKβ were purchased from Cell Signaling Technologies (Boston, MA). NF-κB p65 luciferase reporter plasmid (pNF-κB-Luc) was purchased from Beyotime Institute for Biotechnology (China). Plasmid pCMV-SPORT6 inserted with human RelA sequence driving the expression of p65 was obtained from Thermo Scientific (Rockford, IL). Alexa Fluor 546 conjugated secondary antibody, Lipofectamine 2000, Z’-LYTE™ kinase assay kit and IKKβ recombinant human protein were obtained from Invitrogen-Life Technologies (Carlsbad, CA). Dual-Luciferase Reporter assay kit was obtained from Promega (Madison, WI). Duplex siRNAs with two thymidine residues (dTdT) at the 3′-end of the sequence were synthesized at GenePharma (Shanghai, China). Recombinant human TNF-α was purchased from PeproTech (Rocky Hill, NJ). Unless otherwise stated, all other reagents were obtained from Sigma-Aldrich (St. Louis, MO).

### Compounds

**Figure 1:**
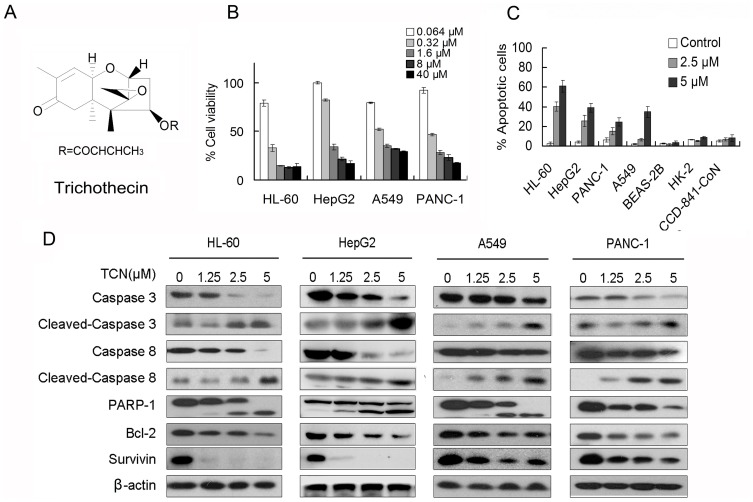
TCN induces apoptosis of NF-κB constitutively activated cancer cells. (A) Chemical structure of trichothecin. (B) Cell cytotoxic effects of trichothecin at successive concentrations. After 48 h treatment, cell viability was determined using MTT assays. (C) Annexin V-FITC/PI analysis of apoptosis in cells treated with TCN for 24 h. (D) HL-60, HepG2, A549 and PANC-1 cells were treated with TCN for 24 h and cell lysates were subjected to western blot analysis with antibodies indicated. β-actin were used as loading controls. Each column represents the mean ± SD of triplicates in three independent experiments.

TCN and other tested compounds were extracted from *Trichothecium roseum* LZ93, an endophytic fungus isolated from *Maytenus hookeri Loes.*, as previously described [24] (structures designated in Figure 1A and the Figure S1A).

### Cytotoxicity Assay and IC_50_ Determination

Cell viability was evaluated by MTT assay. Cells were treated with the indicated compounds at concentrations of 0.064, 0.32, 1.6, 8 and 40 µM in 96-well plates. After 48 h, 0.1 mg MTT was added to each well, for a final concentration of 20%. Cells were then incubated at 37°C for 4 h and the absorbance was measured at 595 nm by spectrophotometry. The IC_50_ values were determined by non-linear regression analysis using GraphPad Prism software (GraphPad, Inc., San Diego, CA).

### Luciferase Reporter Assay

HEK 293T cells were transiently transfected with pNF-κB-Luc and pRL-TK (Promega, Madison, WI) plasmids using Lipofectamine 2000 for 4 h in a 96-well plate. Cells were then pre-incubated with different concentrations of compounds for 1 h, and subsequently activated with 25 ng/mL TNF-α for 18 h. Luciferase activities were measured using the Dual-Luciferase Reporter Assay kit.

### Western Blotting

Total cell lysates were prepared by direct lysis in 2×Laemmli buffer (0.125 M Tris-HCl, pH 6.8, 4% SDS, 20% glycerol, 10% β-mercaptoethanol, and 0.004% bromophenol blue). Samples were then fractionated in 12% acrylamide gel, transferred to a PVDF membrane (Bio-Rad), and incubated with specific primary antibodies followed by the corresponding peroxidase-conjugated secondary antibodies. Proteins of interest were visualized by chemiluminescent detection on an ImageQuant LAS mini4000 (GE Healthcare).

### Immunofluorescence Staining

For p65 translocation experiment, HepG2 cells were grown on chamber slides for 24 h and pre-incubated with TCN at 37°C for 1 h, followed by TNF-α stimulation for 20 min. For IKKβ phosphorylation detection, cells were pre-incubated with TCN at 37°C for 1 h followed by TNF-α stimulation for 10 min. Cells were then fixed in phosphate buffered saline with 4% paraformaldehyde for 20 min, and subsequently permeabilized in phosphate buffered saline with 0.1% Triton X-100. To observe the localization of p65 subunit, cells were incubated with anti-p65 antibody and corresponding FITC conjugated secondary antibody before staining the nuclei with DAPI. For the IKKβ phosphorylation detection, cells were incubated with anti-phospho-IKKβ antibody and corresponding Alexa Fluor 546 secondary antibody. Images were later observed using a fluorescence microscope (Eclipse Ti, Nikon).

### Cell Apoptosis Assay

Cell apoptosis was analyzed using the Annexin V-FITC/PI Apoptosis kit (BD Biosciences, Franklin Lakes, NJ) according to the manufacturer’s protocols. Cells were seeded in 6-well plates at a density of 1.2×10^6^ cells/well. After 24h of compound treatment at the indicated concentrations, cells were collected and then washed twice with cold PBS, and then resuspended in a binding buffer containing Annexin V-FITC and propidium iodine (PI). After incubation for 15 min at room temperature in the dark, the fluorescent intensity was measured using a FACSCalibur flow cytometer (BD Biosciences, Franklin Lakes, NJ).

### Cell Cycle Analysis

HepG2 cells (5×10^5^cells/well in 12-well plates) were incubated with TCN for indicated time points (8, 16 and 24 h) and concentrations (1.25, 2.5 and 5 µM). Cells were collected and washed twice with PBS and were fixed with 70% ethanol overnight. Fixed cells were washed with PBS, and then incubated with 20 µL RNase A (1 mg/mL) for 30 min and stained with propidium iodide (PI) solution (50 µg/mL final concentration) in the dark for 1 h. Fluorescence intensity was analyzed by FACSCalibur flow cytometer (BD Biosciences, Franklin Lakes, NJ). The percentages of the cells distributed in different phases of the cell cycle were determined using FlowJo 7.6.1.

### Overexpression of IKKβ CA

The construct driving the expression of a constitutively active IKKβ (S177E, S181E) (IKKβ CA) was obtained from Addgene (Catalog No.11105) [27]. HepG2 cells were transfected with the plasmids using Lipofectamine 2000. Some 12 h after transfection, cells were treated with TCN for additional 24 h.

### RNA Interference

HepG2 cells were transiently transfected with a scrambled control siRNA (5′-UUCUCCGAACGUGUCACGUTT -3′) and IKKβ-siRNA (5′-GGUGGAAGAGGUGGUGAGCTT -3′) [28], respectively, at a concentration of 150 pmol/60 mm dish using Lipofectamine 2000. The resulting tested compound was later added to the transfected cells 48 h post-transfection.

### IKKβ Kinase Activity Assay

The IKKβ kinase assay was performed with Z’-LYTE™ Kinase Assay kit following the manufacturer’s protocols. In brief, recombinant human IKKβ proteins were incubated with the kinase reaction mixture with increasing amounts of TCN or staurosporine, a kinase inhibitor, for 1h. The reaction was then stopped with the stop reagent and the fluorescence was detected with excitation at 400 nm and emissions at 445 nm and 520 nm using the EnVision Multimode Plate Reader.

## Results

### TCN Inhibits Cell Growth and Induces Apoptosis in NF-κB Constitutively Activated Cancer Cells

We investigated the anticancer activity of TCN by MTT cell viability assay in four cancer cell lines harboring constitutively activated NF-κB [29]–[32]. In all tested cell lines, TCN exhibited obvious growth inhibition (Figure 1B). The IC_50_ values in HL-60, HepG2, A549 and PANC-1 cells were 0.18, 0.82, 0.39, and 0.28 µM, respectively. Annexin V-FITC/PI staining was further analyzed for cell apoptosis with flow cytometry. After treatment with 5 µM TCN for 24 h, cell apoptosis in HL-60, HepG2, A549 and PANC-1 cells remarkably elevated to 61.13%, 44.03%, 34.93%, and 24.47%, respectively. Meanwhile, apoptosis in human normal cell lines BEAS-2B, HK-2 and CCD-841-CoN were not affected by TCN treatment, even at the highest concentration 5 µM, suggesting TCN possesses selectivity against cancer cells (Figure 1C). Western blot analysis also demonstrated that TCN significantly induced the activation of caspase-8 and caspase-3, as well as the cleavage of PARP-1 in the four cancer cell lines. The protein level of Bcl-2, a key regulator of the intrinsic apoptotic pathway, was also down-regulated by TCN treatment. Moreover, the level of survivin, an anti-apoptotic protein, dramatically decreased in TCN treated cells, especially in HL-60 and HepG2 cells (Figure 1D).

### TCN Inhibits NF-κB Signaling and Induces Cell Cycle Arrest at G0/G1

**Figure 2:**
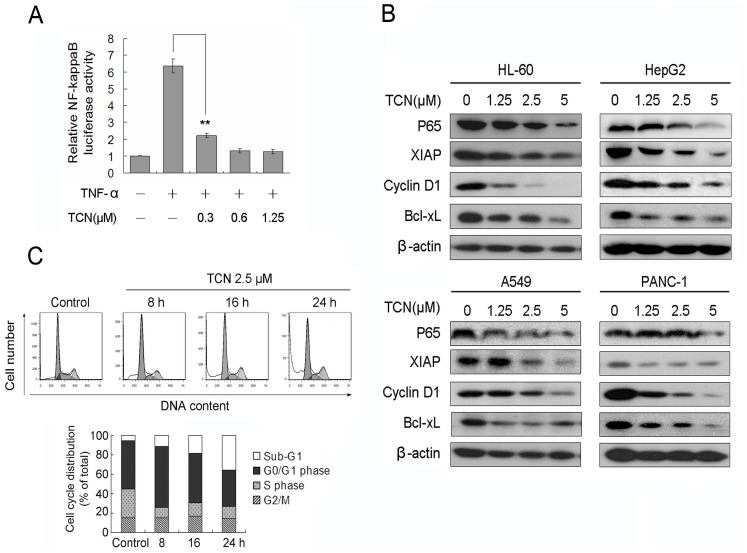
TCN inhibits NF-κB signaling and induces cell cycle arrest. (A) HEK 293T cells were transiently transfected with pNF-κB-Luc plasmids followed by treatment with TCN for 1 h before being stimulated with 25 ng/mL TNF-α for 18 h. (B) Lysates fromcells treated with TCN for 24 h were subjected to western blot analysis with p65, XIAP, cyclin D1 and Bcl-xL antibodies. (C) HepG2 cells were treated with 2.5 µM TCN for 8, 16 and 24 h. Cells were harvested and subjected to cell cycle analysis. The percentage of cells of different phases of cell cycle was analyzed by FlowJo. Experiments were done independently in triplicate, results are reported as means and standard deviations. Statistical significance was analyzed by One-way ANOVA, **p<0.01.

The effect of TCN on NF-κB signaling was tested in HEK 293T cells transiently transfected with an NF-κB reporter (pNF-κB-Luc).The reporter gene expression was clearly activated by TNF-α, which was efficiently inhibited by TCN (Figure 2A). To check whether TCN inhibits the intrinsic NF-κB in cancer cells, we studied the expression of NF-κB target genes in the four NF-κB activated cancer cell lines treated with TCN. The protein levels of p65, XIAP, cyclin D1, and Bcl-xL were clearly down-regulated in TCN treated cells (Figure 2B). Since Cyclin D1 is required for G1/S cell cycle progression, we investigated the effect of TCN on the cell cycle arrest. HepG2 cells were treated with 2.5 µM TCN. Obvious G0/G1 cell cycle arrest was detected as early as 8h. However, with prolong of the treatment, increase of cells of Sub-G1 phase were observed, indicating cell apoptosis induced by TCN (Figure 2C).

We further investigated the effects of trichothecolone, a derivative of TCN, on cell growth and NF-κB activity. Trichothecolone is another metabolite isolated from endophytic fungus of *Maytenus hookeri Loes.*, which shares the same parent nucleus with TCN, with the notable exception of a different substituent at 4-OH [24]. Like TCN, trichothecolone also inhibited cell growth and NF-κB reporter activity (Figure S1), but both activities are much weaker, suggesting that the substituent at 4-OH of this class of compounds is critical for their biological activities.

### TCN Inhibits the Phosphorylation and Degradation of IκBα and Blocks p65 Nuclear Translocation

**Figure 3:**
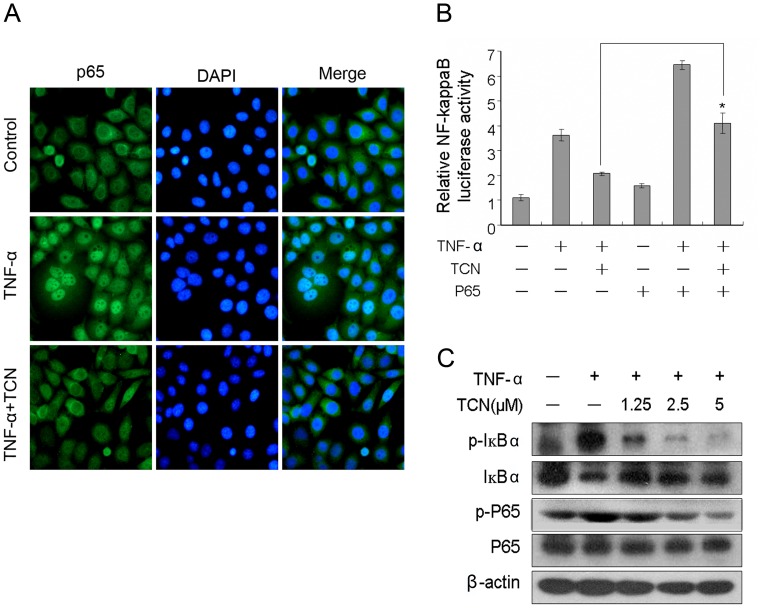
TCN blocks TNF-α induced p65 nuclear translocation and the phosphorylation of IκBα. (A) HepG2 cells pretreated with 2.5 µM TCN were stimulated with 25 ng/mL TNF-α for 20 min and processed for immunostaining with anti-p65 antibody. Nuclei of cells were stained with DAPI (blue) and p65 was visualized by green fluorescence. (B) HEK293T cells were transiently transfected with pNF-κB-Luc and p65 expression plasmids followed by pretreatment of 0.3 µM TCN and stimulation with 25 ng/mL TNF-α. Reporter activity was then measured. (C) HepG2 cells pretreated with TCN were collected after stimulation with 25 ng/mL TNF-α for 10 min. Cell lysates were then analyzed by western blot using antibodies against phospho-IκBα, phospho-p65 and IκBα. Experiments were done independently in triplicate and the results are reported as means and standard deviations. Statistical analysis was perform with Student’s t-test, *p<0.05.

We checked the effect of TCN on the nuclear translocation of p65, one hallmark of the activation of NF-κB signaling. TNF-α treatment induced the translocation of NF-κB from the cytoplasm to the nucleus, and TCN significantly blocked the translocation process in HepG2 cells (Figure 3A). Overexpression of p65 markedly reversed the inhibitory effect of TCN on the transcription of the NF-κB reporter (Figure 3B). We then tested the effect of TCN on IκBα phosphorylation and degradation. TCN inhibited TNF-α induced IκBα phosphorylation in a dose-dependent manner and markedly blocked the degradation of IκBα. More poignantly, we found that TCN inhibited p65 phosphorylation at Ser536 (Figure 3C), which contributes to the degradation of IκBα and the activation of NF-κB signaling [33], [34].

### TCN Inhibits the Phosphorylation of IKKβ Induced by TNF-α

**Figure 4:**
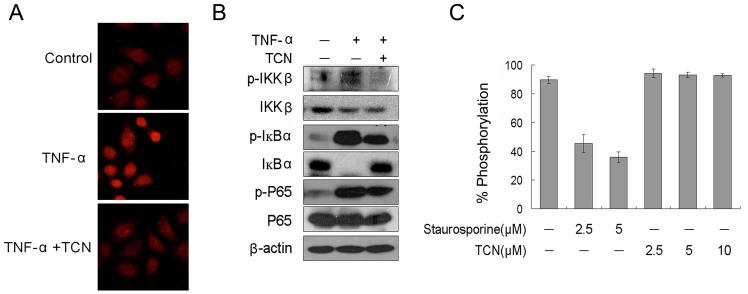
TCN inhibits the phosphorylation of IKKβ. (A) IKKβ phosphorylation was detected by phospho-IKKβ antibody in HepG2 cells stimulated with 25 ng/mL TNF-α for 10 min. Cells were fixed, permeabilized, and examined by fluorescence microscope. (B) Western blot analysis showing the inhibition of IKKβ phosphorylation in cells treated with 2.5 µM TCN. Cells were collected after stimulated with 25 ng/mL TNF-α for 10 min. (C) IKKβ kinase activity was analyzed with recombinant IKKβ using the Z’-LYTE™ kinase assay kit. Fluorescence was detected at 400 nm for the excitation wavelengths and 445 nm and 520 nm for the emission wavelengths. Experiments were done independently in triplicate and the results are reported as means and standard deviations.

As IκBα and p65 are both substrates of IKKβ kinase, which is activated by undergoing phosphorylation (Ser 177 and Ser 181) and subsequently phosphorylates IκBα [35], [36], we checked the phosphorylation of IKKβ in TNF-α treated HepG2 cells by immunofluorescence and western blot analysis. We found that the level of phosphorylated IKKβ dramatically decreased following TCN treatment, with the total IKKβ protein level unaltered (Figure 4A and B). To determine whether TCN interferes with the IKKβ kinase activity directly, a kinase activity assay was carried out using purified human IKKβ recombinant protein using staurosporine, a potent kinase inhibitor, as the positive control. The result showed that TCN did not affect the kinase activity of recombinant IKKβ (Figure 4C). These data demonstrated that TCN inhibits NF-κB signaling by suppressing the activation of IKKβ via blocking its phosphorylation.

### TCN Induced Cancer Cell Apoptosis is Mediated by Inhibition of IKKβ Phosphorylation

**Figure 5:**
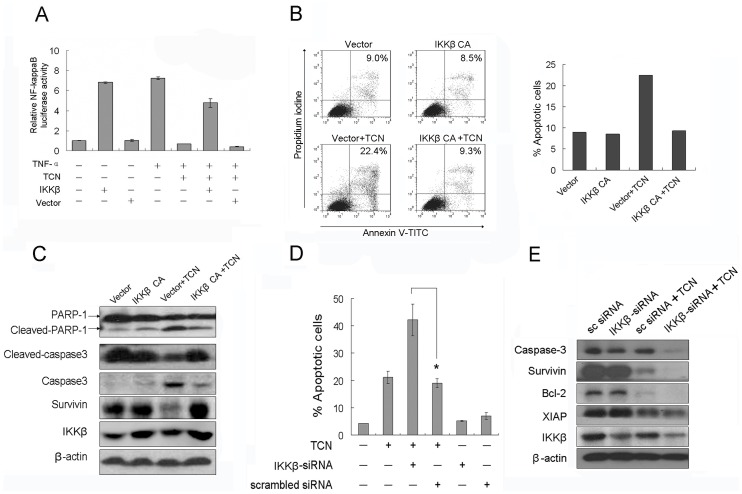
TCN induced cancer cell apoptosis is mediated by inhibition of IKKβ phosphorylation. (A) HEK 293T cells were transiently transfected with IKKβ CA or empty vector for 12 h, then pretreated with 2.5 µM TCN and stimulated with 25 ng/mL TNF-α for 18 h. Cells subjected to analysis of luciferase activity. (B) HepG2 cells were transiently transfected with IKKβ CA or empty vector for 12 h, and then treated with 2.5 µM TCN for 24 h. Cells subjected to apoptosis analysis. (C) HepG2 cells were transfected with IKKβ CA or empty vector for 12 h, and then treated with 2.5 µM TCN for 24 h. Cells subjected to western blot analysis for the expression of indicated proteins. (D) HepG2 cells were transiently transfected with a scrambled siRNA or IKKβ-siRNA for 48 h, treated with 2.5 µM TCN for 24 h and subjected to apoptosis analysis. (E) HepG2 cells transiently transfected with scrambled siRNA or IKKβ-siRNA were treated with 2.5 µM TCN for 24 h. Cell lysates were collected and subjected to western blot analysis with the specified antibodies. Experiments were done independently in triplicate and the results are reported as means and standard deviations. Statistical analysis was perform with Student’s t-test, *p<0.05.

TCN induced cell death was investigated in cells overexpressing constitutively active (CA) form of IKKβ [27], [28]. As shown in Figure 5A, overexpression of IKKβ CA in HepG2 cells sufficiently activated NF-κB signaling in luciferase activity assay. TCN treatment antagonized with TNF-α in activation of NF-κB signaling, whereas IKKβ CA transfection efficiently reversed the inhibitory effect of TCN. Consistently, overexpression of IKKβ CA aborted the TCN induced cell apoptosis in HepG2 cells (Figure 5B). Meanwhile, western blot analysis showed the deactivation of caspase-3, PARP-1 and upregulation of survivin by IKKβ CA (Figure 5C).

Next, we checked the effect of IKKβ knockdown on the apoptotic activity of TCN. Compared with treatment with TCN alone, knockdown of IKKβ with siRNA sensitized HepG2 cells to TCN-induced apoptosis, with the apoptotic ratio increasing to 43.11% (18.17% in TCN treatment alone) (Figure 5D). As shown in Figure 5E, the expression of IKKβ in HepG2 was partly decreased by the treatment of IKKβ siRNA. Consistent with the increased cell apoptosis induction, down-regulation of the anti-apoptotic proteins (survivin, XIAP and Bcl-2) by TCN was enhanced in IKKβ knockdown cells, and the cleavage of caspase-3 was increased as well (Figure 5E). Meanwhile, transfection with a control scrambled siRNA had no effect on the response of HepG2 cells to TCN treatment (Figure 5D, 5E).

## Discussion

Given the connection between NF-κB and cancer, the development of NF-κB inhibitor holds great potential in suppressing certain types of cancer proliferation as well as improving existing cancer therapies. Over the past few decades, a diverse variety of natural and synthetic compounds have been found capable of suppressing NF-κB signaling, but through a variety of different mechanisms. PS-341, a proteasome inhibitor, has been a first-line drug used in treating multiple myeloma for over a decade, which works by inhibiting intrinsic NF-κB activity by blocking IκBα degradation [37]. Meanwhile, Eriocalyxin B, an ent-Kauranoid isolated from *Isodon eriocalyx*, inhibits NF-κB activation by interfering with the binding of both p65 and p50 to the response element [38].

IKKβ plays central role in the activation of both canonical and non-canonical NF-κB signaling pathway. Upon activation, NF-κB signaling leads to auto-polyubiquitination of tumor necrosis factor receptor associated factor 6 (TRAF6). The ubiquitinated TRAF6 then recruits the transforming growth factor-β-activated kinase 1 (TAK1) and the IκB kinase (IKK) complex, which consists of two catalytic subunits, IKK1 (IKKα) and IKK2 (IKKβ), and a regulatory subunit, NEMO (NF-κB essential modulator, IKKγ), so that TAK1 can phosphorylate and activate IKKβ. The auto-phosphorylation also was reported to be involved in the activation of IKKβ [39], [40]. Due to frequently observed role that the activation of IKKβ plays in cancer generation, proliferation and metastasis, IKKβ has been considered a promising drug target for cancer treatments [41], [42]. An FDA approved orphan drug to treat pancreatic cancer, CDDO-Me, also known as RTA 402, blocks the NF-κB pathway through direct inhibition of IKKβ on Cys-179 [43].

In the current study, we described TCN, a potent NF-κB inhibitor, blocked IKKβ activation by suppressing its phosphorylation and subsequently inhibited the expression of the target genes of NF-κB signaling pathway, including XIAP, cyclin D1 and Bcl-xL, which regulate cell survival and cell proliferation. As a result, TCN induced cell apoptosis and cell cycle arrest in NF-κB constitutively activated human cancer cells, without affecting normal cells with low basal NF-κB activity (Figure S2). Considering the complex events associating with the activation of IKKβ, the precise mechanisms how TCN impairs the phosphorylation of IKKβ are worthy of further investigation.

Metabolites of endophytic fungi colonizing in herbal medicine have been found to possess various bioactivities [44], [45]. Anticancer agents of many host plants are found in metabolites of the endophytic fungi, such as Taxol, which was isolated from endophytic fungus *Taxomyces andreanae* in *Taxus brevifolia* [46]. In the present study, TCN, along with 6β-hydroxyrosenonolactone, trichothecolone, roseocardin and roseotoxin B were isolated from endophytic fungus LZ93 of *Maytenus hookeri Loes.* and were tested for their anticancer activities (Figure S1). Among the compounds we isolated, TCN proved to be the most potent. These findings indicate that properties of TCN might be one of the potential mechanisms underlying the efficacy and anti-cancer activities of *Maytenus hookeri Loes.*

Taken on the whole, our findings suggest that TCN, as a potent inhibitor of NF-κB signaling, has promising therapeutic value for cancer treatment and deserves further exploration.

## Supporting Information

Figure S1 Bio-activities of compounds isolated from endophytic fungus LZ93 of *Maytenus hookeri Loes.*
(A) Chemical structures of 6β-hydroxyrosenonolactone (6β-HRL), trichothecolone, roseocardin and roseotoxin B. (B) Cytotoxic effects induced by trichothecin, trichothecolone, 6β-hydroxyrosenonolactone, roseocardin and roseotoxin B at 40 µM in HL-60, HepG2, A549 and PANC-1 cells after 48 h treatment. (C) Effect of trichothecin, trichothecolone, 6β-hydroxyrosenonolactone, roseocardin and roseotoxin B on TNF-α-induced NF-κB activation. HEK 293T cells were transiently transfected with pNF-κB-Luc and pRL-TK plasmids followed by pretreatment with DMSO, or 0.3, 0.6, 1.25 µM TCN, or successive concentrations of 2.5, 5, 10 µM of trichothecolone, 6β-hydroxyrosenonolactone, roseocardin or roseotoxin B for 1 h before 25 ng/mL TNF-α stimulation for 18 h. Progressively darker shading of each bar indicates higher concentrations.
(JPG)

Figure S2 Schematic diagram of TCN inhibition of IKKβ and the NF-κB pathway. Upon stimulated by TNF-α, a panel of kinases will undergo ubiquitination and phosphorylation, which results in activation of NF-κB via IKKβ medicated degradation of IκBα and translocation of p65. TCN inhibits the phosphorylation of IKKβ, which in turn results in apoptosis and growth inhibition in cancer cells.
(TIF)
