# Supplementary figures and images for: Trichothecin Induces Cell Death in NF-κB Constitutively Activated Human Cancer Cells via Inhibition of IKKβ Phosphorylation
Source: PLoS One. 2013 Aug 1;8(8):e71333. doi: 10.1371/journal.pone.0071333 (PMC3731298; doi:10.1371/journal.pone.0071333)

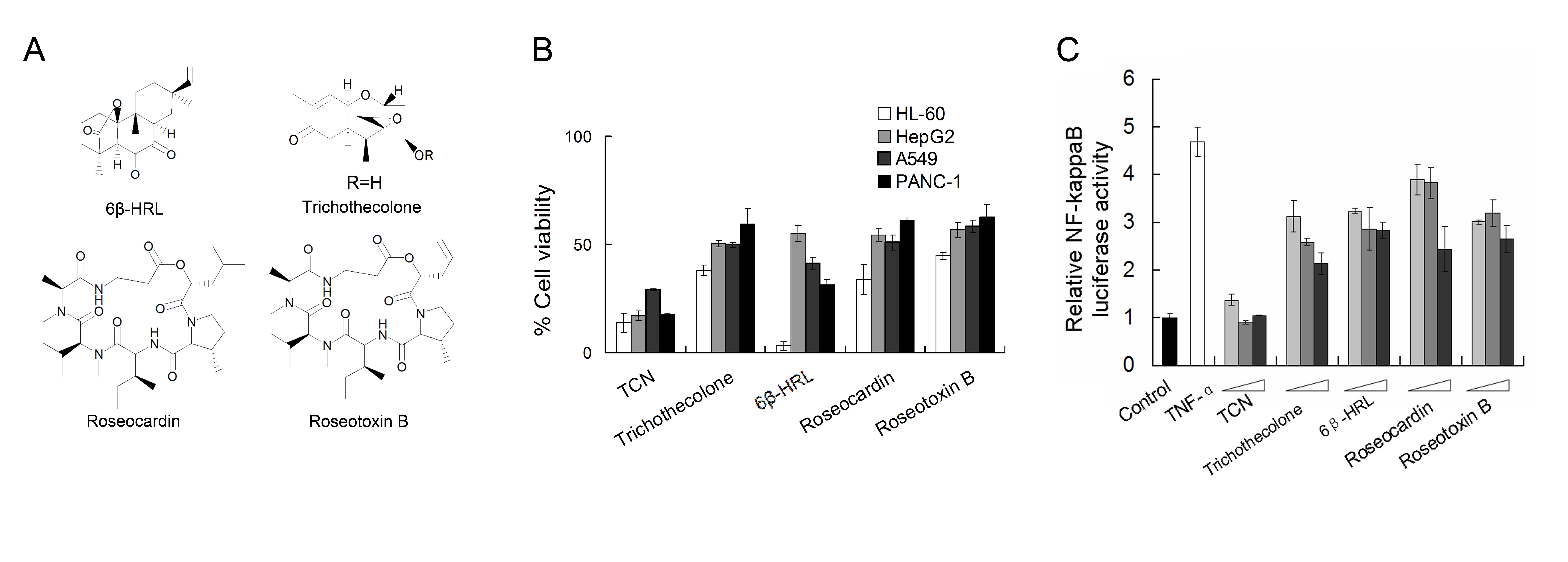

Supplement: Figure S1 — (A) Chemical structures of 6β-hydroxyrosenonolactone (6β-HRL), trichothecolone, roseocardin and roseotoxin B. (B) Cytotoxic effects induced by trichothecin, trichothecolone, 6β-hydroxyrosenonolactone, roseocardin and roseotoxin B at 40 µM in HL-60, HepG2, A549 and PANC-1 cells after 48 h treatment. (C) Effect of trichothecin, trichothecolone, 6β-hydroxyrosenonolactone, roseocardin and roseotoxin B on TNF-α-induced NF-κB activation. HEK 293T cells were transiently transfected with pNF-κB-Luc and pRL-TK plasmids followed by pretreatment with DMSO, or 0.3, 0.6, 1.25 µM TCN, or successive concentrations of 2.5, 5, 10 µM of trichothecolone, 6β-hydroxyrosenonolactone, roseocardin or roseotoxin B for 1 h before 25 ng/mL TNF-α stimulation for 18 h. Progressively darker shading of each bar indicates higher concentrations. (JPG) [file pone.0071333.s001.jpg]

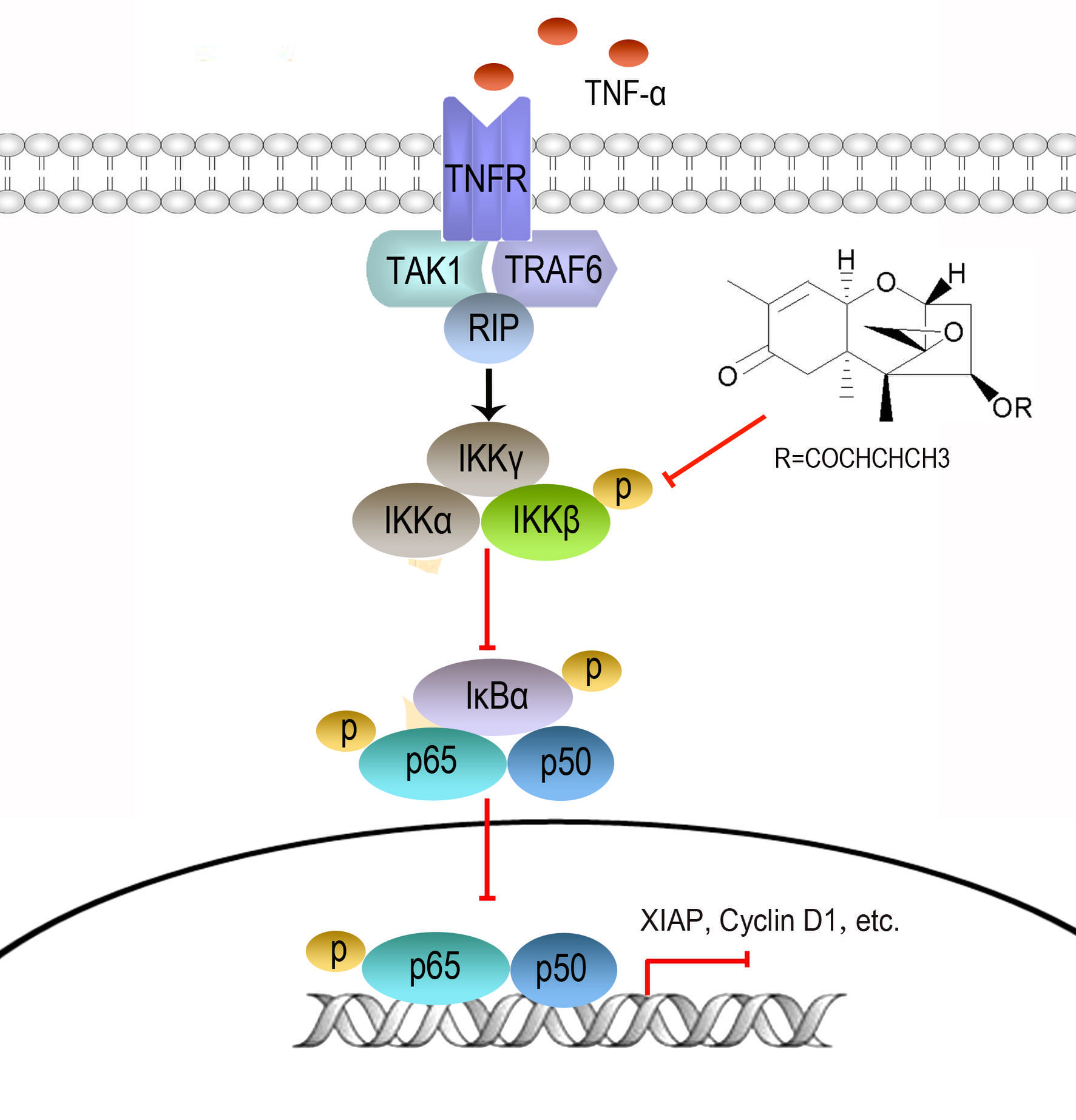

Supplement: Figure S2 — Upon stimulated by TNF-α, a panel of kinases will undergo ubiquitination and phosphorylation, which results in activation of NF-κB via IKKβ medicated degradation of IκBα and translocation of p65. TCN inhibits the phosphorylation of IKKβ, which in turn results in apoptosis and growth inhibition in cancer cells. (TIF) [file pone.0071333.s002.tif]
